# Non-invasive identification of brain signatures of acute liver injury

**DOI:** 10.7150/thno.127621

**Published:** 2026-01-21

**Authors:** Santhoshi P. Palandira, Aidan Falvey, Joseph Carrion, Qiong Zeng, Saher Chaudhry, Kira Grossman, Lauren Turecki, Leann Mahadeo, Nha Nguyen, Michael Brines, Christine N. Metz, Yousef Al-Abed, Sangeeta S. Chavan, Eric H. Chang, Yilong Ma, David Eidelberg, An Vo, Kevin J. Tracey, Valentin A. Pavlov

**Affiliations:** 1 The Feinstein Institutes for Medical Research, Northwell Health, Manhasset, NY, USA.; 2 Elmezzi Graduate School of Molecular Medicine, 350 Community Drive, Manhasset, NY 11030, USA.; 3 Donald and Barbara Zucker School of Medicine at Hofstra/Northwell, Hempstead, NY, USA.

**Keywords:** acute liver injury, microPET imaging, brain dysfunction, neuroinflammation, brain energy metabolism, brain metabolic connectivity, diagnosis

## Abstract

**Background:** In many disorders, metabolic and inflammatory derangements that originate in peripheral organs have a deleterious impact on the brain. Brain functional impairment, defined as *hepatic encephalopathy,* is one of the main diagnostic criteria for acute liver failure (ALF), a severe complication of acute liver injury (ALI). While brain inflammation (neuroinflammation) and metabolic alterations significantly contribute to hepatic encephalopathy, their non-invasive evaluation remains challenging.

**Methods:** To address this limitation, we utilized dual radiotracer [^18^F]-fluoro-2-deoxy-2-D-glucose ([^18^F]FDG) and [^11^C]-peripheral benzodiazepine receptor ([^11^C]PBR28) microPET imaging followed by conjunction analysis and metabolic connectivity mapping. We applied this advanced methodology in mice with high dose acetaminophen (N-acetyl-p-aminophenol, APAP)-induced ALI, which can progress into ALF.

**Results:** We observed hepatocellular damage, liver and systemic inflammation, and increased density of hippocampal microglia in mice with ALI. MicroPET imaging analysis characterized the presence of brain region-specific neuroinflammation and altered brain energy metabolism in mice with ALI. We also identified both gains and losses in connectivity, as well as a dual role of neuroinflammation. These results revealed brain “neuroinflammetabolic” signatures of ALI.

**Conclusion:** These findings provide a platform for non-invasively diagnosing early signs of hepatic encephalopathy with the goal of informing timely diagnoses and targeted therapies. Our approach can be further utilized in non-invasive brain assessments in liver diseases and other disorders classically characterized by peripheral immune and metabolic dysregulation.

## Introduction

The brain is affected, sometimes severely, in many disorders primarily driven by peripheral metabolic and inflammatory pathologies, including acute and chronic liver diseases 1-9. Acute liver injury (ALI) is a life-threatening condition that frequently leads to hospitalization and is the most common identifiable cause of acute liver failure (ALF) 10-12. Brain dysfunction and neurological complications are common in ALI/ALF. These complications are defined as *hepatic encephalopathy,* which is a major diagnostic criterion for ALF 2, 13. There is a correlation between developing hepatic encephalopathy during ALF and increased mortality 2, 14. Among several factors in the brain, neuroinflammation (inflammation in the CNS) significantly contributes to hepatic encephalopathy in ALF 3, 14-17. The neuroinflammatory alterations in ALF advance in parallel with the progression of hepatic encephalopathy 1, 3, 15, 18. In addition, alterations in brain energy metabolism during ALF have been implicated in the pathogenesis of hepatic encephalopathy 1, 17. Therefore, non-invasive evaluation of neuroinflammatory alterations and changes in brain energy metabolism in the context of ALI/ALF is of specific interest for early diagnosis and therapeutic interventions.

A reliable non-invasive technique for brain neuroinflammatory and metabolic evaluation is provided by positron emission tomography (PET) brain imaging using specific radiotracers. The radiotracer [^11^C]-peripheral benzodiazepine receptor ([^11^C]PBR28) has been extensively used for PET evaluation of neuroinflammation in rodents 19, 20, non-human primates 21, and humans 22-25. [^18^F]-fluoro-2-deoxy-2-D-glucose ([^18^F]FDG) is a radiotracer widely utilized to determine alterations in brain energy metabolism using PET 26, 27. We recently developed a dual tracer, [^11^C]PBR28 and [^18^F]FDG approach for brain microPET imaging with conjunction analysis 28. Using this approach, we identified brain regions with overlapping neuroinflammatory changes and alterations in energy metabolism in mice with systemic inflammation induced by peripheral endotoxin administration 28.

Here, we apply our methodology to evaluate the brain in mice with ALI, which progresses into ALF. At least 45% and 65% of all ALF cases that occur in the United States and the United Kingdom, respectively, and about 20% of all liver transplant cases are related to overdose with acetaminophen (APAP, Tylenol) 13, 29-31, one of the most widely used medications worldwide. Therefore, we utilize a mouse model of APAP induced ALI manifested by sustained hepatocellular damage and other characteristic metabolic and inflammatory alterations. Using conjunction analysis of PET imaging data, we revealed the presence of characteristic neuroinflammatory and metabolic alterations in the hippocampus, thalamus, and other brain regions. In addition, we applied an advanced method for brain metabolic connectivity analysis. This method has been previously utilized to identify alterations in brain networks i.e., brain disease-related functional topographies in patients with neurodegenerative diseases and other disorders 32. Using this method for the first time in rodents, we identified specific changes in brain metabolic connectivity in mice with ALI. These findings reveal characteristic brain “neuroinflammetabolic” signatures of ALI. They support the use of this PET imaging-based approach as a platform for brain evaluation during liver diseases and other disorders with peripheral organ origin, with the goal of timely diagnosis and treatment.

## Materials and Methods

### Animals

Experiments were performed in accordance with the National Institutes of Health guidelines, and all experimental procedures with animals were approved by the Institutional Animal Care and Use Committee and the Institutional Biosafety Committee of the Feinstein Institutes for Medical Research. C57BL/6J male mice (13-15 weeks old) were obtained from the Jackson Laboratory and were maintained at 25 °C on a 12-h light/dark cycle with free access to food and water.

### APAP-induced ALI in mice and subsequent experiments

After an overnight (16 h) fast, mice were injected intraperitoneally (i.p.) with 600 mg/kg acetaminophen (APAP, Sigma, St. Louis, MO) dissolved in 10% DMSO in sterile saline or vehicle (10% DMSO in sterile saline, vehicle). Vehicle (control) and APAP administered mice were euthanized at 24 h or 48 h, and blood was collected via cardiac puncture for serum aminotransferase and IL-6 measurements. The liver was collected for histological analysis following 4% paraformaldehyde-PBS transcardiac perfusion and IL-6 determination by ELISA after homogenizing the tissue or by histology (see below for details). Perfusion was also performed post-cardiac puncture followed by liver removal. A pump was inserted into the intact left ventricle and the mice were perfused with 20 mL of ice-cold PBS, followed by 20 mL of 4% paraformaldehyde. Whole brains were extracted from the skull and placed in 10 mL of 4% paraformaldehyde for 24 h and then transferred to 10 mL of 30% sucrose and processed for hippocampal IBA1 immunostaining. Other cohorts of vehicle and APAP injected mice underwent microPET imaging 24 h and 48 h post-injection. Following each microPET scan, mice were euthanized.

### Liver histological analysis and quantification

Following perfusion, the liver from vehicle- and APAP-treated mice was collected, and liver tissue was fixed with 4% paraformaldehyde in PBS, followed by paraffin-embedding before sectioning. Liver sections were stained with hematoxylin-eosin (H&E) and examined for hepatocellular damage as previously reported 33, 34. Quantitative analysis of the extent of tissue injury was performed after digitally imaging three high-power fields per slide in a random and blinded fashion. Areas of tissue injury were identified based on necrotic alterations, involving loss of cellular architecture, cell disruption, and vacuolization. The necrotic areas were quantified using Fiji Image processing software. The average necrotic area percentage from each animal was used for subsequent analysis.

### Serum aminotransferase analysis

Serum levels of alanine aminotransferase (ALT) and aspartate aminotransferase (AST) activity were measured in mice 24 h and 48 h post-APAP or vehicle-treatment using commercial assay ALT and AST kits (Sigma-Aldrich Co. LLC) following the manufacturers' recommendations.

### Serum IL-6 determination

At 24 h and 48 h post vehicle or APAP injection, blood was collected via cardiac puncture and centrifuged; serum was collected and stored at -80°C for IL-6 analysis by ELISA. At the same time points, livers were collected and immediately processed in tissue protein extraction reagent (ThermoFisher Scientific). Liver lysates were assessed using a standard Bradford protein assay kit (Bio-Rad). Subsequently serum and liver tissue IL-6 levels were quantified using an IL-6 ELISA kit (DY406, R&D Systems), according to the manufacturer's directions. Serum IL-6 levels were expressed as picograms per milliliter (pg/mL) of serum collected and hepatic IL-6 levels were expressed as picograms per mg (pg/mg) hepatic protein.

### Brain processing, IBA1 immunohistochemistry, and quantification

Brains were sectioned on a vibratome at 50 µm. 12 sections were collected from the beginning of the hippocampal region of bregma -1.55 as defined by the Paxinos and Franklin mouse brain atlas (i.e. -1.55 to -2.15). Following a standardized 'free-floating sections' protocol, sections were washed, blocked (10% bovine serum albumin) and then a rabbit anti-IBA1 primary antibody was incubated (1/400) with the sections for three days at 4 ^°^C. Subsequently, the sections were washed and incubated with a fluorescent (647: 1/500) donkey anti-rabbit secondary antibody for two days at 4 ^0^C. Sections were again washed and then incubated with DAPI (1/10000) prior to mounting onto microscope slides. Images were captured on the Zeiss LSM 880 confocal microscope for the broad regional structure of the right and left hippocampus at 20x; as well as 40x for the CA2 region of the hippocampus. A direct count of microglia was performed per field of view within the broad regional structure of the right and left hippocampus: the count was averaged across multiple replicates and each section had their area standardized for the variations between bregma (-1.55 - -2.15).

### MicroPET imaging and analysis

MicroPET imaging was performed using the Inveon® MicroPET imaging system (Siemens) at 24 h or 48 h post vehicle or APAP administration. Briefly, upon arrival in the imaging suite, animals were acclimated for 1 h and then anesthetized with 2-2.5% isoflurane mixed in oxygen, positioned head-to-head with a custom-made isoflurane nose cone on the camera gantry, and the tail vein cannulated using a 30 G custom catheter. [^11^C]PBR28 and [^18^F]FDG are routinely synthesized onsite at the PET radiochemistry department at the Feinstein Institutes and delivered directly into the microPET suite. After laser cross-hair centering in the field of view (FOV), approximately 0.5 mCi of [^11^C]PBR28 (in 0.2 mL) was slowly injected via the tail vein (i.v.) into each animal with the simultaneous start of a 60-min dynamic imaging acquisition (10 x 1 min, 5 x 2 min, 2 x 5 min, and 15 x 2 min), followed by a 10-min transmission scan for attenuation correction. 1.5 h after the end of the [^11^C]PBR28 scan, ~0.5 mCi of [^18^F]FDG (in 0.3 mL) was injected i.p. with 35-40 mins allowed for uptake of the tracer, followed by a 10 min static emission scan and a 10 min transmission scan. Brain images were reconstructed using Inveon Acquisition workflow (IAW 1.5) and three-dimensional ordered subsets-expectation maximization (3D-OSEM) reconstruction with attenuation correction using the corresponding PET transmission scan for each tracer (given that the animals were immobile on the gantry over the course of imaging acquisition). After reconstruction, raw PET images were bounding box aligned, skull stripped, and dose and weight corrected using Pixel-Wise Modeling (PMOD) 4.0 Software. [^18^F]FDG scans from each animal were registered to an [^18^F]FDG brain template 35, with the same rigid transformations applied to the corresponding [^11^C]PBR28 scans. All registered images for each animal were then spatially normalized via [^18^F]FDG scans to a common MRI brain template 36, which was aligned within Paxinos and Franklin anatomical space 37, using statistical parametric mapping (SPM) mouse toolbox within MATLAB. For each [^11^C]PBR28 scan, the final 10 frames (i.e., the last 20 min of the dynamic scan) were averaged and used for analysis. Images were smoothed with an isotropic Gaussian kernel FWHM (full width at half maximum) of 0.56 mm at all directions.

To identify regions in anatomical space with significant differences between APAP and vehicle administered mice in both [^18^F]FDG and [^11^C]PBR28 tracers at 24 h and 48 h, we performed whole-brain voxel-wise searches with conjunction analysis using SPM-Mouse software (The Wellcome Centre for Human Neuroimaging, UCL Queen Square Institute of Neurology, London, UK, https://www.fil.ion.ucl.ac.uk/spm/ext/#SPMMouse) 38. The conjunction analysis identified the group effects common to both radiotracers, in which the contrasts, testing for a group effect, were specified separately for each tracer. These contrasts were set at a common threshold and combined to give the conjunction result. The combination was on a voxel-by-voxel basis, and a new contrast that tests for the conjunction was created. This model was set up with full factorial analysis (2x2) with 2 tracers ([^18^F]FDG and [^11^C]PBR28) and 2 groups (APAP and vehicle). Inter-subject variability in imaging data was accounted for by dividing each PET scan by its global mean value.

Brain metabolic connectivity analysis was performed using grouped data from [^18^F]FDG microPET scans of APAP-treated and control mice at 24 h and 48 h. The mouse brain was parcellated into 28 bilateral regions of interest (56 nodes, see **Table S1**) using the Waxholm atlas 39. For each node, we computed normalized metabolic activity based on [^18^F]FDG PET scans. The data from each group were utilized to construct node-to-node correlation matrices separately for the vehicle and APAP-treated groups. We generated 100 bootstrap samples for each group, calculating pairwise nodal Pearson correlation coefficients for each iteration. The median values of the 100 bootstrap correlation estimates were used to create an adjacency matrix for the [^18^F]FDG network in APAP-treated and control mice. These calculations were performed using the Machine Learning Toolbox in MATLAB R2024b. We examined changes in metabolic connectivity, reported as gains or losses between the two groups, by comparing all the connection pairs using a previously described method 40. A connection was considered gained or lost relative to a reference if either the ALI or the control group exceeded |*r*| ≥ 0.6 (*P* < 0.05) and |*∆r*| > 0.4 (*P* < 0.05, permutation test, 1000 iterations). Validation of connections meeting these criteria was performed using 100 bootstrap samples (*P* < 0.05, Student's t-test).

### Statistical analysis

All data are presented as the mean ± SEM. Statistical analyses of experimental data were conducted using GraphPad Prism 9.5.0 (GraphPad Software, Inc., La Jolla, CA). After evaluating the data for normality using a Shapiro-Wilk test, differences between two groups were assessed using an unpaired two-tailed Student's t test (for normally distributed data) or the Mann-Whitney test (when the data did not meet the assumptions of normality). Histological liver injury data were analyzed using the non-parametric Kolmogorov-Smirnov test. In post-hoc analysis of microPET data, values for each significant cluster were similarly compared between the two groups using the unpaired Mann-Whitney test. *P ≤* 0.05 was considered significant.

In statistical analysis of imaging data, group differences were considered significant at a voxel-level threshold of *P <* 0.01 for conjunction analysis and individual analysis with a cluster cutoff of 200 voxels. We identified the significant conjunction clusters, in which both [^18^F]FDG and [^11^C]PBR28 values increased or decreased in APAP-administered mice relative to vehicle-administered mice. We checked if there were significant clusters, in which values were increased in one tracer and decreased in the other in APAP-treated relative to vehicle-treated mice or vice versa. We also performed whole-brain voxel-wise searches separately for each tracer to validate the results from the conjunction analysis. Individual data from each significant cluster (in Paxinos and Franklin anatomical space) 37 were identified throughout the whole-brain searches and were measured with post-hoc volume-of-interest (VOI) analyses using in-house MATLAB scripts. [^18^F]FDG and [^11^C]PBR28 values for the APAP- and vehicle-treated mice were visualized to evaluate overlapping data and potential outlier effects. The TrackVis software (http://www.trackvis.org/) was used to create three-dimensional (3D) visualizations that highlight significant regions in both the conjunction analysis for the two tracers and the individual analysis for each tracer.

## Results

### APAP administration results in significant ALI and increased IL-6 levels at 24 and 48 h

**Figure 1:**
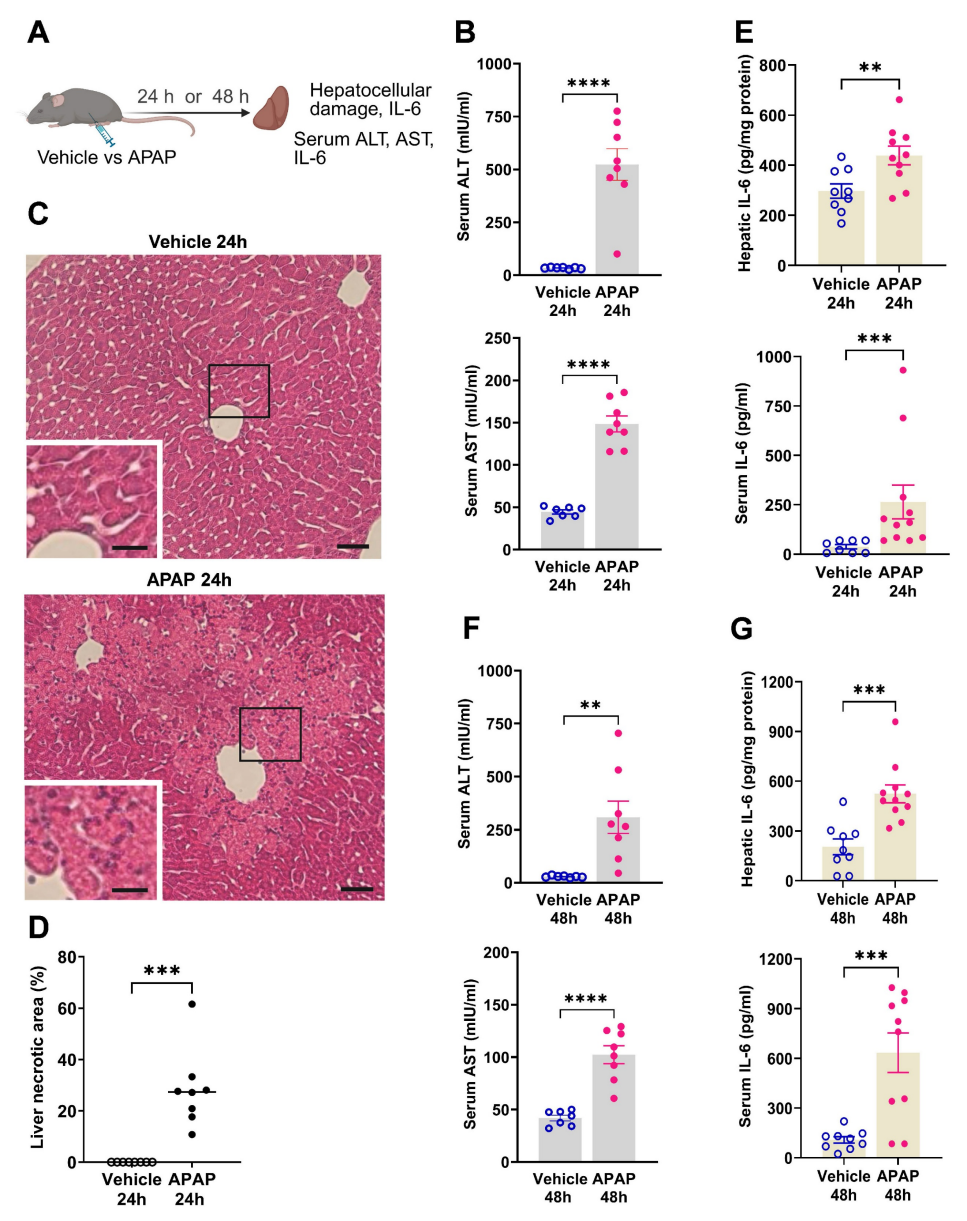
** APAP induces significant liver injury and increases hepatic and serum IL-6 levels. (A)** Vehicle or APAP was injected (i.p.), and hepatic and circulating indices were analyzed 24 h or 48 h later. (**B**) Serum ALT and AST levels are significantly higher in APAP-treated (n = 8) compared with vehicle-treated mice (n = 7) (****P < 0.0001; Student's *t*-test) at 24 h.** (C)** APAP induces hepatocellular damage at 24 h, including loss of cellular integrity and vacuolization as indicated in representative images (scale bar = 50 µm in the main images and scale bar = 25 µm in the inserted images). **(D)** Quantification of liver injury based on necrotic areas of liver slices (***P = 0.0002; Kolmogorov-Smirnov test) (n = 8). (**E**) Hepatic IL-6 levels (**P = 0.009; Student's *t*-test) and serum IL-6 levels (***P = 0.0002; Mann-Whitney test) are significantly increased in APAP-mice (n = 10,11) compared with controls (n = 8,9) at 24 h.** ( F)** Serum ALT and AST levels are significantly higher in APAP administered mice (n = 8) compared with vehicle treated controls (n = 7) (**P = 0.005, ****P < 0.0001; Student's *t*-test) at 48 h. (**G**) Hepatic (***P = 0.0004; Student's *t*-test) and serum IL-6 levels (***P = 0.0007; Student's *t*-test) are significantly increased in APAP mice (n = 10,11) compared with controls (n = 9) at 48 h. Data are presented as individual mouse data points with mean ± SEM.

We first characterized the severity of ALI in mice injected with APAP vs. vehicle (**Figure 1A**). APAP administration resulted in significantly elevated ALT and AST levels compared with vehicle (control) treatment at 24 h post treatment (**Figure 1B**). Consistent with these results, histological evaluation at 24 h revealed the presence of liver injury in APAP-administered mice. As illustrated in representative images/photomicrographs of H&E-stained liver sections, there were areas with relatively large spans of hepatocellular necrosis, indicated by a loss of cellular architecture and vacuolization in APAP-treated mice at 24 h (**Figure 1C**). No such histological alterations were observed in H&E-stained liver sections from vehicle-treated mice (**Figure 1C**). Quantitative analysis demonstrated the significant hepatocellular damage in APAP-treated mice compared with vehicle treated controls (**Figure 1D**). As previously reported APAP (600 mg/kg) administration in C57Bl/6 mice causes sustained necrotic hepatocellular injury demonstrated at 48 h, utilizing histological H&E staining and quantitative analysis 33. These findings and the significantly elevated ALT and AST levels in APAP-treated mice compared with controls at 48 h (**Figure 1F**) rendered it unnecessary to perform histological evaluation of the liver injury at 48 h in the two groups of mice. In ALI, hepatocellular damage is accompanied by enhanced circulating cytokine levels and inflammation, with increased serum IL-6 levels previously implicated in mediating brain alterations during high-dose APAP-induced ALI 41. Accordingly, we analyzed and determined significantly elevated hepatic and serum cytokine IL-6 levels in mice treated with APAP vs. vehicle at 24 h (**Figure 1E**) and 48 h (**Figure 1G**). These results demonstrate significant ALI and both hepatic and systemic inflammation at 24 h and 48 h following high dose APAP administration.

### ALI is associated with microglial alterations in the hippocampus at 24 and 48 h

**Figure 2:**
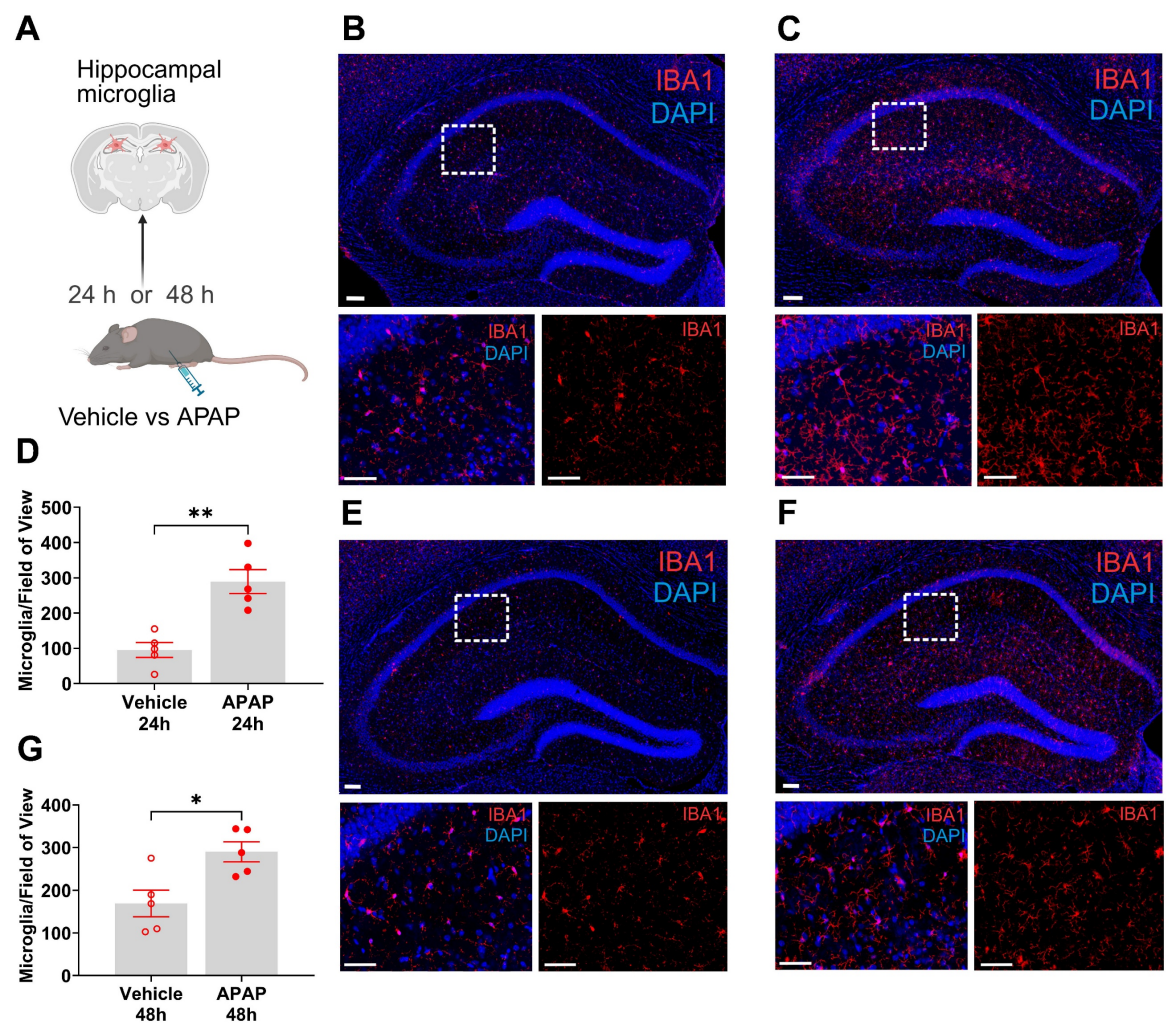
** APAP induced liver injury is associated with significant increases of hippocampal microglia.** (**A**) Vehicle or APAP was injected (i.p.), and hippocampal microglia analysis was performed at 24 h and 48 h. (**B**) Representative images of hippocampal IBA1 positive cells in vehicle treated mice at 24 h. Low magnification image (top, scale bar = 100 μm) with boxed area magnified and presented below (bottom, scale bar = 50 μm). (**C**) Representative images of hippocampal IBA1-positive cells in APAP treated mice at 24 h. 20 X magnification image (top, scale bar = 100 μm) with boxed area that is magnified and presented below (bottom, scale bar = 50 μm). (**D**) Microglial quantitation based on IBA1 staining demonstrating a significant increase in the hippocampus of APAP-treated mice (n = 4) compared with vehicle treated controls (n = 5) (**P = 0.001, Student's *t*-test). Data are presented as individual mouse data points with mean ± SEM. (**E**) Representative images of hippocampal IBA1-positive cells in vehicle treated mice at 48 h. 20 X magnification image (top, scale bar = 100 μm) with boxed area that is magnified and presented below (bottom, (scale bar = 50 μm). (**F**) Representative images of hippocampal IBA1-positive cells in APAP treated mice at 48 h. Low magnification image (top, scale bar = 100 μm) with boxed area that is magnified and presented below (bottom, scale bar = 50 μm). (**G**) Microglial quantitation based on IBA1 staining demonstrating significant increase in the hippocampus of APAP-treated mice (n = 5) compared with vehicle treated controls (n = 5) (*P = 0.015, Student's *t*-test). Data are presented as individual mouse data points with mean ± SEM.

Microglia are cells with key immune functions in the brain 42. Microglia-neuron interactions also play a key role in maintaining neuronal integrity and function 42, 43. Increased systemic inflammation and peripheral metabolic derangements have been linked to brain, including microglial alterations 28, 44, 45. Increased microglia density and other alterations have been associated with neuroinflammation and disruption of the physiological microglia-neuron relationship and impaired neuronal function 46-48. The hippocampus is a brain area in which microglial alterations have been documented during aberrant systemic inflammation and increased circulatory levels of IL-6 and other cytokines 47, 48. We have previously demonstrated hippocampal microglial alterations following peripheral endotoxin administration in mice in parallel with increased circulating IL-6 and other cytokine levels 45. Here, we examined hippocampal microglia in mice with APAP induced ALI compared with vehicle treated controls at 24 h and 48 h using IBA1 immunolabeling (**Figure 2A**). We observed a significant increase in the density of microglia in mice treated with APAP, indicative of neuroinflammatory activation (**Figure 2B-D**). Similarly, 48 h post vehicle injection, there was a significant increase in the number of microglia within the broad regional structure of the hippocampus (**Figure 2E-G**). These results demonstrate the presence of increased microglial density in the hippocampus at 24 h and 48 h during ALI.

### ALI causes significant brain neuroinflammatory and metabolic alterations at 24 h

**Figure 3:**
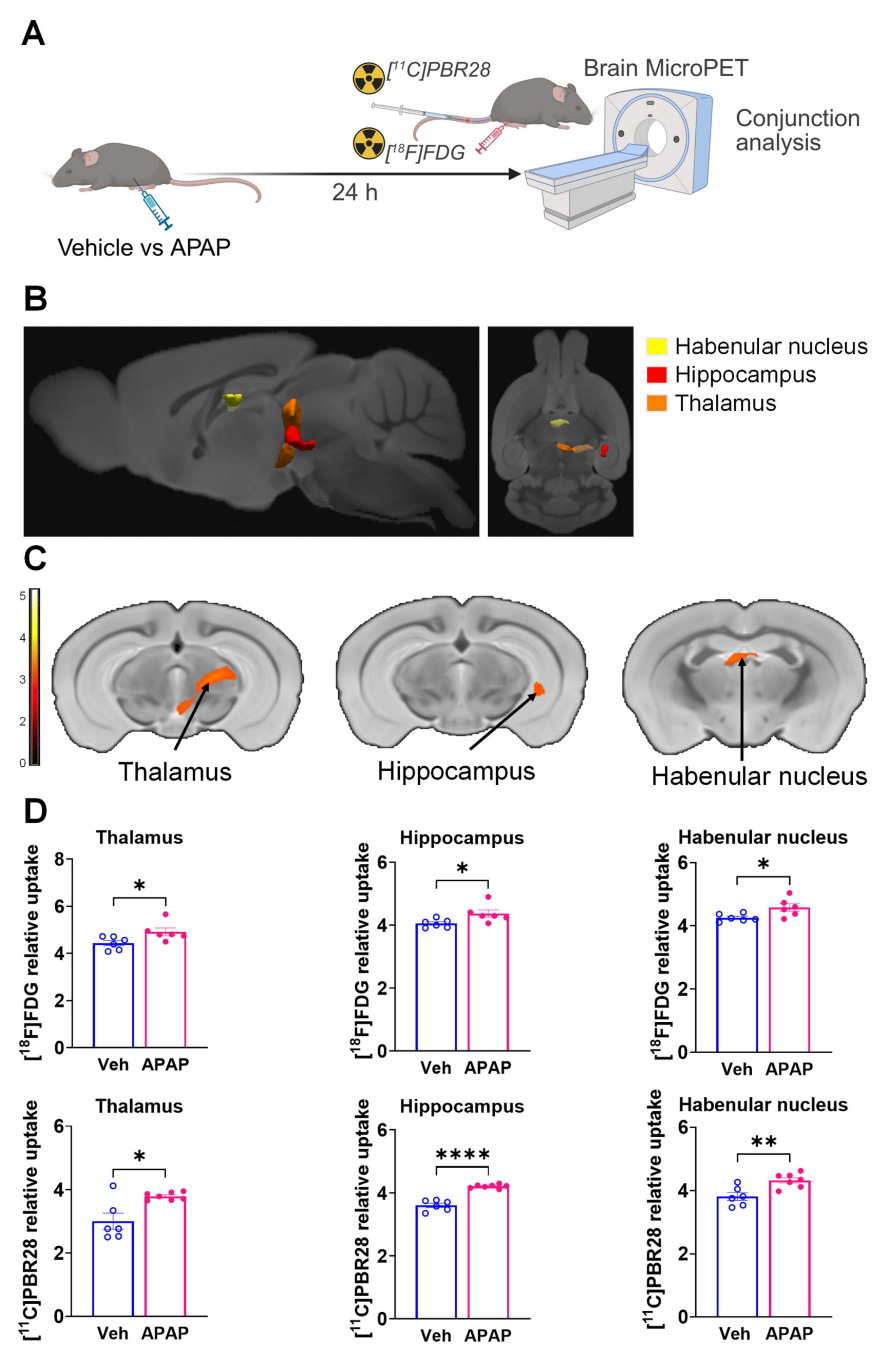
** Brain regions with overlapping [^11^C]PBR28 and [^18^F]FDG uptake increases at 24 h during ALI.** (**A**) Vehicle or APAP was injected (i.p.) in mice, followed by radiotracer ([^11^C]PBR28, i.v., tail vein, and [^18^F]FDG, i.p.) administration and microPET imaging 24 h later, and conjunction analysis. (**B**) Cumulative dual tracer uptake increases (statistically significant clusters at *P <* 0.01) in the thalamus, the hippocampus, and the habenular nucleus in APAP administered vs control mice as 3D clusters overlaid on brain sagittal and axial MRI templates; and (**C**) overlaid on brain coronal MRI templates (color bar represents t-value height, cutoff threshold T = 2.4). See **Table S2** for stereotaxic coordinates. (**D**) Post-hoc analysis for the same statistically significant increases in [^18^F]FDG and [^11^C]PBR28 uptake in vehicle (control) (n = 6) and APAP-treated mice (n = 6, 7). Statistically significant clusters show overlapping regions of increased uptake of [^18^F]FDG in the thalamus (*P = 0.036; Student's t test), the hippocampus (*P = 0.023; Mann-Whitney test), and the habenular nucleus (*P = 0.026; Mann-Whitney test) as well as [^11^C]PBR28 in the thalamus (*P = 0.047; Mann-Whitney test), the hippocampus (****P < 0.0001; Student's t test), and the habenular nucleus (**P = 0.005; Student's t test) in APAP administered vs control mice. Data are presented as individual mouse data points with mean ± SEM.

**Figure 4:**
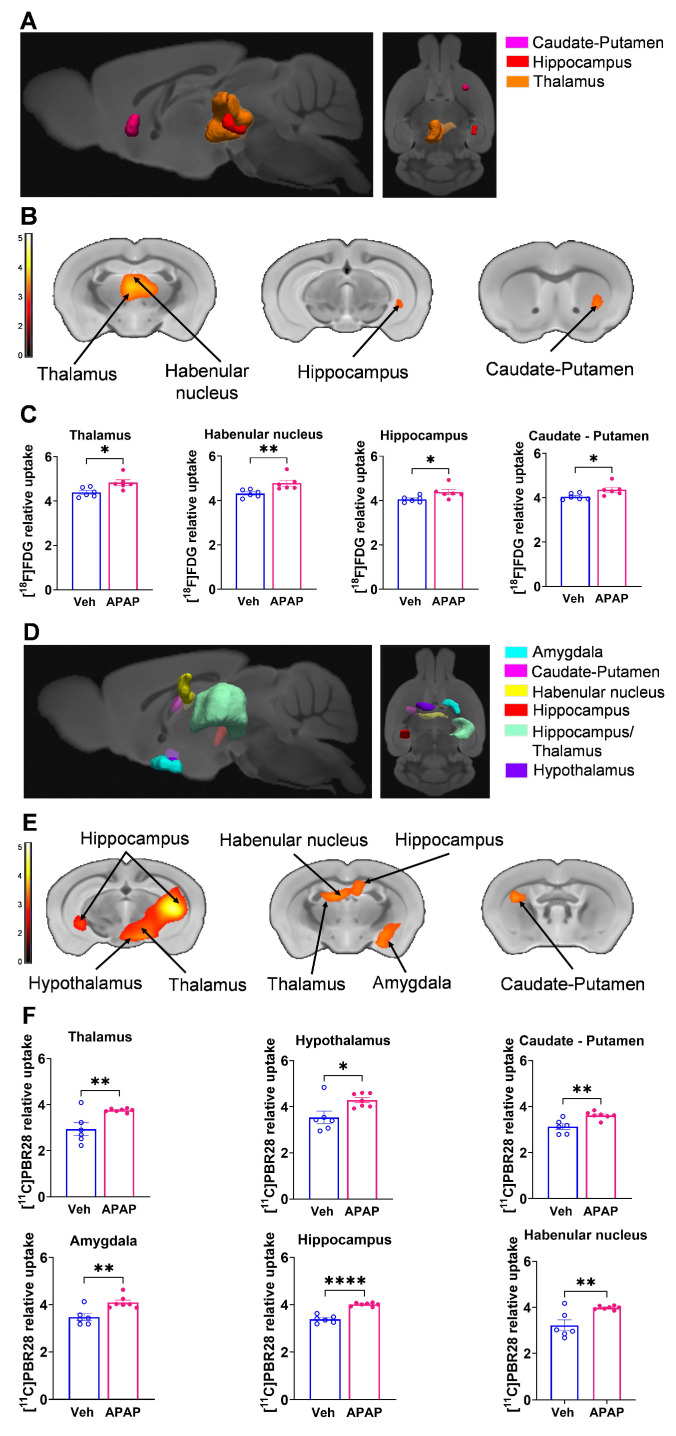
** Brain regions with individual [^11^C]PBR28 and [^18^F]FDG uptake increases at 24 h during ALI.** (**A**) Cumulative [^18^F]FDG uptake increases (statistically significant clusters at *P <* 0.01) in the thalamus, the hippocampus, the habenular nucleus, and the caudate-putamen in APAP administered vs control mice as 3D clusters overlaid on brain sagittal and axial MRI templates; and (**B**) overlaid on brain coronal MRI templates (color bar represents t-value height, cutoff threshold T = 2.4). See **Table S2** for stereotaxic coordinates. (**C**) Post-hoc analysis of [^18^F]FDG tracer uptake in the thalamus (*P = 0.016; Student's *t*-test), habenular nucleus (**P = 0.002; Mann-Whitney test), hippocampus (*P = 0.037; Student's *t*-test), and caudate-putamen (*P = 0.029; Student's *t*-test) (n = 6). (**D**) 3D clusters of cumulative increase in [^11^C]PBR28 uptake (*P <* 0.01) in the thalamus, the hippocampus, the habenular nucleus, the hypothalamus, the amygdala, and the caudate putamen in APAP administered vs control mice. (**E**) Cumulative increases in [^11^C]PBR28 uptake (*P <* 0.01) on brain coronal MRI brain templates (color bar represents t-value height, cutoff threshold T = 2.4). See **Table S2** for stereotaxic coordinates. (**F**) Post-hoc analysis of [^11^C]PBR28 uptake in the thalamus (**P = 0.008, Student's *t*-test), the hypothalamus (*P = 0.023, Student's *t*-test), the caudate putamen (**P = 0.0035; Student's *t*-test), the amygdala (**P = 0.004; Student's *t*-test), the hippocampus (****P < 0.0001; Student's *t*-test). and the habenular nucleus (**P = 0.005; Student's *t*-test), (n = 6,7). Data are presented as individual mouse data points with mean ± SEM.

Our results, demonstrating peripheral metabolic and inflammatory derangements and increased brain microglia density in mice with APAP-induced ALI prompted us to perform further non-invasive brain evaluation. MicroPET imaging utilizing [^11^C]PBR28 (~ 0.5 mCi), followed by [^18^F]FDG (0.5 mCi) was acquired in mice injected with vehicle or APAP 24 h earlier (**Figure 3A**). We first utilized a conjunction analysis to identify brain areas with significantly increased simultaneous uptake of [^11^C]PBR28 and [^18^F]FDG in mice 24 h following APAP administration compared with vehicle administration. Applying this analysis revealed simultaneous (overlapping) increases in the uptake of both tracers in the thalamus, the hippocampus, and the habenular nucleus of mice administered with APAP. These overlapping cumulative increases are presented as 3D clusters on sagittal and transverse MRI templates in **Figure 3B** and highlighted on brain coronal section templates (**Figure 3C**). Additional post-hoc analysis of these clusters revealed the magnitude of these simultaneous increases (**Figure 3D**). Next, we performed individual analyses of [^18^F]FDG and [^11^C]PBR28 brain uptake in APAP administered vs control mice. In line with results from the conjunction analysis, we observed significant [^18^F]FDG uptake increases in the thalamus, hippocampus, and habenular nucleus. In addition, a significant [^18^F]FDG uptake increase was demonstrated in the caudate-putamen (**Figure 4A-C**). [^11^C]PBR28 brain uptake was significantly higher in the thalamus, hypothalamus, hippocampus, caudate-putamen, and amygdala of APAP-administered mice compared with vehicle injected controls (**Figure 4D-F**). The SPM conjunction and individual analyses identified specific patterns of increased brain [^18^F]FDG and [^11^C]PBR28 uptake, as shown in **Table S2**. Applying conjunction analysis indicated no decreases in tracer uptake (APAP < control) in any brain region. However, individual analysis demonstrated that [^18^F]FDG uptake was significantly lower in the primary motor and the primary somatosensory cortex. In addition, [^11^C]PBR28 uptake was significantly decreased in the cerebellum, while a marginal decrease was detected in the primary somatosensory cortex (P = 0.061) (**Figure S1A-F**). Of note, no brain regions were showing joint decreases in [^18^F]FDG uptake and increases in [^11^C]PBR28 uptake or vice versa in APAP mice compared with vehicle injected controls. Together, these results define the presence of neuroinflammation and altered brain metabolic homeostasis in mice with APAP-induced ALI at 24 h.

### ALI results in brain neuroinflammatory and metabolic changes at 48 h

**Figure 5:**
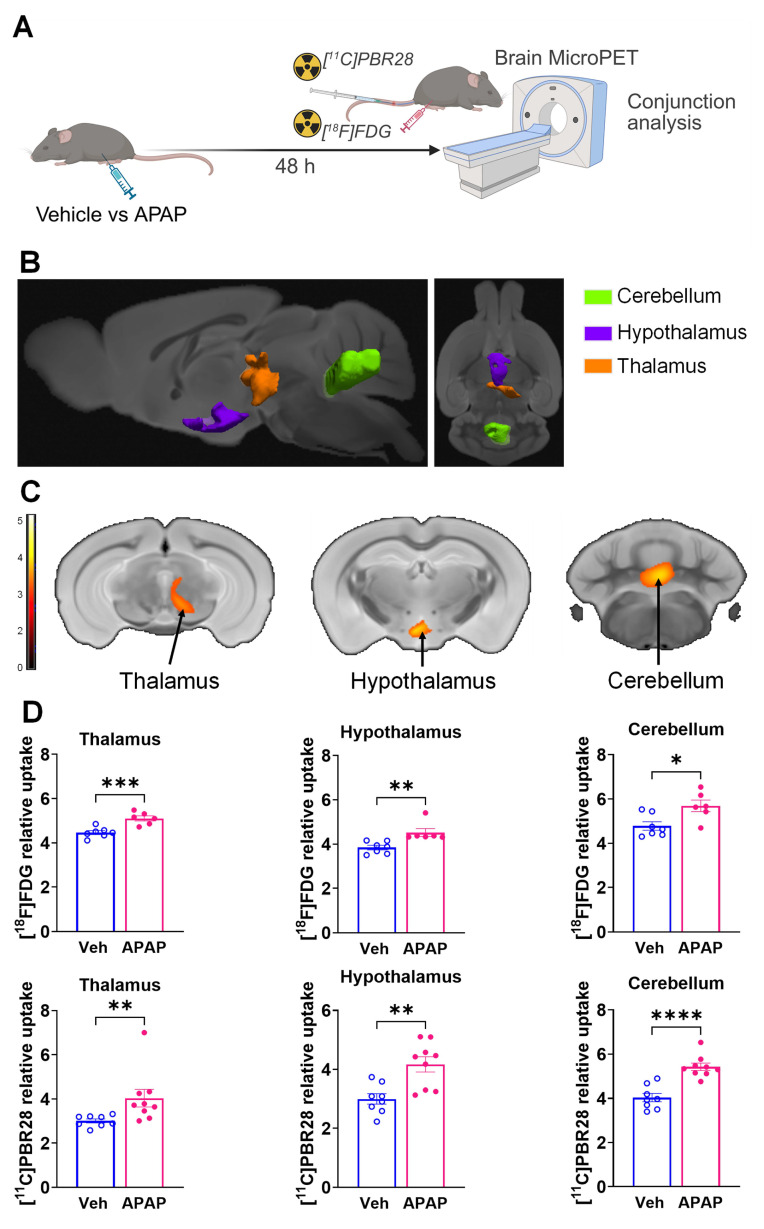
** Brain regions with overlapping [^11^C]PBR28 and [^18^F]FDG uptake increases at 48 h during ALI.** (**A**) Vehicle or APAP was injected (i.p.) in mice, followed by radiotracer ([^11^C]PBR28, i.v., tail vein, and [^18^F]FDG, i.p.) administration and microPET imaging 48 h later, and conjunction analysis. (**B**) Cumulative dual tracer uptake increases (statistically significant clusters at *P <* 0.01) in the thalamus, the hypothalamus and the cerebellum in APAP administered vs control mice as 3D clusters overlaid on brain sagittal and axial MRI templates; and (**C**) overlaid on brain coronal MRI templates (color bar represents t-value height, cutoff threshold T = 2.4). See **Table S3** for stereotaxic coordinates. (**D**) Post-hoc analysis for the same statistically significant increases in [^18^F]FDG uptake in the thalamus (***P = 0.0008; Student's *t*-test), hypothalamus (**P = 0.001; Mann-Whitney test) and the cerebellum (*P = 0.015; Student's *t*-test) as well as [^11^C]PBR28 uptake in the thalamus (**P = 0.006; Mann-Whitney test), hypothalamus (**P = 0.002; Students *t*-test) and the cerebellum (****P < 0.0001; Student's *t*-test) in control (n = 7,8) and APAP mice (n = 6,9). (For one vehicle treated mouse and three APAP treated mice only [^11^C]PBR28 (and no [^18^F]FDG) image acquisition was achieved and used in the analysis at 48 h) Data are presented as individual mouse data points with mean ± SEM.

**Figure 6:**
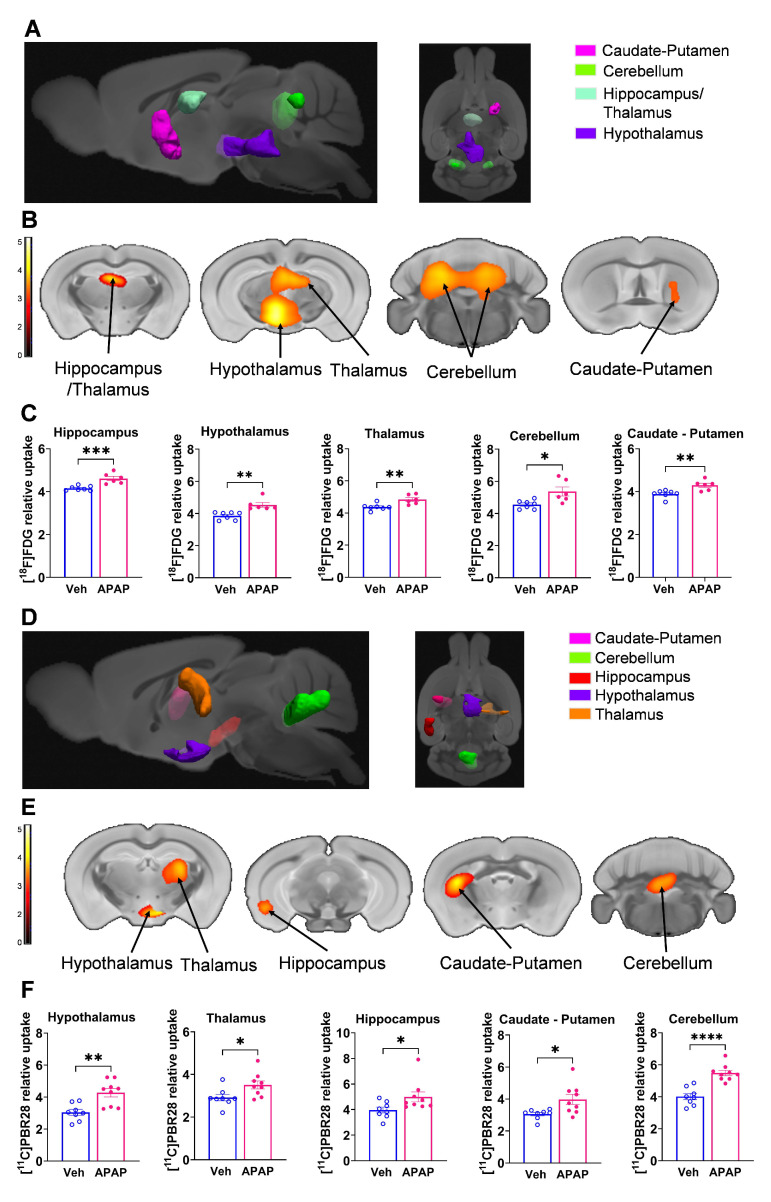
** Brain regions with individual [^11^C]PBR28 and [^18^F]FDG uptake increases at 48 h during ALI.** (**A**) Cumulative [^18^F]FDG uptake increases (statistically significant clusters at *P <* 0.01) in the thalamus, the hippocampus, the hypothalamus, the caudate-putamen, and the cerebellum as 3D clusters overlaid on brain sagittal and axial MRI templates; and (**B**) overlaid on brain coronal MRI templates (color bar represents t-value height, cutoff threshold T = 2.4). See **Table S3** for specific stereotaxic coordinates. (**C**) Post-hoc analysis of [^18^F]FDG tracer uptake in the hippocampus (***P = 0.0009; Student's *t*-test), hypothalamus (**P = 0.001; Mann-Whitney test), thalamus (**P = 0.006; Students *t*-test), cerebellum (*P =0.014; Student's *t*-test) and the caudate-putamen (**P = 0.005; Student's *t*-test) (n = 7,6). (**D**) 3D clusters of cumulative increase in [^11^C]PBR28 uptake (*P <* 0.01) in the thalamus, the hypothalamus, the hippocampus, the caudate-putamen, and the cerebellum in APAP administered vs control mice. (**E**) Cumulative increase in [^11^C]PBR28 uptake (*P <* 0.01) on brain coronal MRI brain templates (color bar represents t-value height, cutoff threshold T = 2.4). See **Table S3** for stereotaxic coordinates. (**F**) Post-hoc analysis of [^11^C]PBR28 uptake in the hypothalamus (**P = 0.002; Student's *t*-test), thalamus (*P = 0.028; Student's *t*-test), hippocampus (*P = 0.027; Mann-Whitney test), caudate-putamen (*P = 0.018; Student's *t*-test) and the cerebellum (****P < 0.0001; Student's *t*-test) (n = 8,9). Data are presented as individual mouse data points with mean ± SEM.

Using microPET brain imaging, we next investigated the scope of brain neuroinflammatory and metabolic alterations in mice treated with APAP compared with vehicle injected mice at 48 h (**Figure 5A**). First, applying conjunction analysis, we observed significantly higher simultaneous uptake of [^11^C]PBR28 and [^18^F]FDG in the thalamus, hypothalamus, and cerebellum. These brain changes are shown in 3D on sagittal and axial MRI templates (**Figure 5B**) and brain coronal section templates (**Figure 5C**). The post-hoc analysis revealed the exact magnitude of these statistically significant alterations (**Figure 5D**). These increases were accompanied by higher individual [^18^F]FDG (**Figure 6A-C**) and [^11^C]PBR28 (**Figure 6D-F**) uptakes in the same brain regions of APAP treated mice vs vehicle administered controls. Furthermore, region-specific increases for each tracer were identified; as [^18^F]FDG as well as [^11^C]PBR28 uptake was increased in the hippocampus and in the caudate-putamen of mice treated with APAP. The regions with shared increases in [^18^F]FDG and [^11^C]PBR28 uptake are presented in **Table S3**. Shared reductions in tracer uptake were seen in the primary somatosensory cortex of APAP treated mice compared with controls as shown in **Figure S2A-C.** These decreases in the simultaneous uptake were in line with the identified individual decreases in each tracer in the same brain region as shown in **Figure S3A-F.** Of note, in contrast to areas with increases or decreases in the uptake of both tracers, there was a decrease in the [^18^F]FDG uptake and an increase in the [^11^C]PBR28 uptake in the piriform cortex of APAP administered mice as shown in **Figure S4A-C**. These results indicate significant region-specific brain alterations indicative of neuroinflammation and altered energy metabolism in mice 48 h after the onset of APAP-induced ALI.

### The brain metabolic connectivity is altered during ALI

**Figure 7:**
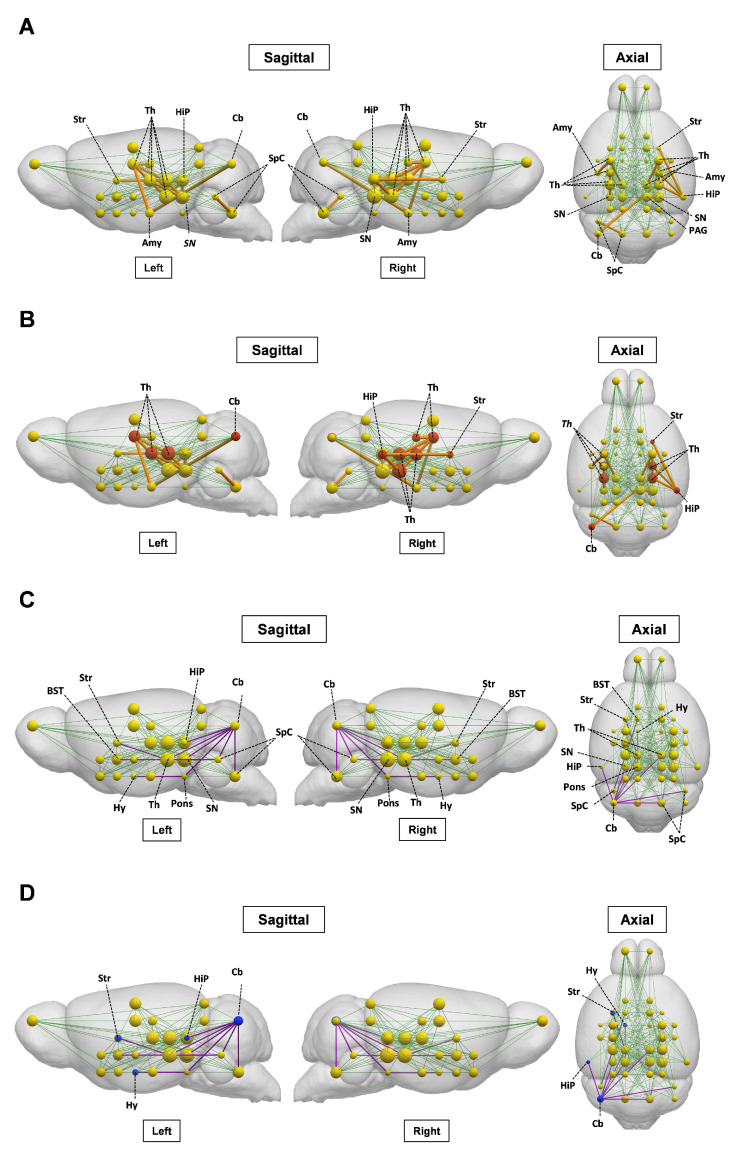
** Brain network alterations during ALI.** (**A**) Metabolic [^18^F]FDG gained connectivity in mice with ALI relative to control mice. [^18^F]FDG regions of interest (nodes) are shown as yellow spheres, with the radius of each node proportional to the degree centrality, which is the number of connections. Normal network connectivity is represented by green lines. Significant gains in connectivity between nodes are represented by orange lines, with thickness proportional to connectivity strength. (**B**) Regions of interest with [^18^F]FDG gained connectivity and neuroinflammatory ([^11^C]PBR28) increases (in red). (**C**) Metabolic [^18^F]FDG lost connectivity in mice with ALI relative to control mice. The normal network connections are represented by green lines. Significant connectivity loses are shown with purple lines. (**D**) Regions of interest with [^18^F]FDG lost connectivity and neuroinflammatory ([^11^C]PBR28) increases (in blue). (vehicle n = 13 and APAP n = 12) Abbreviations: Sensorimotor cortex/Neocortex, S1/M1; Brainstem, SpC; Thalamus, Th; Superior Colliculus (Midbrain), SC; Inferior Colliculus, IC; Periaqueductal gray, PAG; Septal nuclei/Limbic-frontal, Sept; Ventral thalamic nuclei/Thalamus, Th; Pontine nuclei/Pons, Pons; Substantia nigra/Midbrain, SN; Interpeduncular nucleus/Midbrain, MBr-u (unsegmented); Globus pallidus, GP; Mesenphalic nuclei/Midbrain, MBr-u; Laterodorsal nucleus/Thalamus, Th; Medial geniculate nucleus/Thalamus, Th; Anterior pretectal nucleus/Midbrain, MBr-u (unsegmented); Caudate putamen, Str; Hippocampus, HiP; Lateral geniculate nucleus/Thalamus, Th; Amygdala, Amg; Hypothalamus, Hy; Nucleus accumbens/Striatum, Str; Cochlear nuclei/Brainstem, SpC; Cerebellum, Cb; Piriform cortex, PIR, Preoptic area/anterior hypothalamus, Hy; Bed nucleus stria terminalis/extended amygdala, BST; Ventral Pallidum, PALv

We next investigated the impact of ALI on the pre-established ([^18^F]FDG) defined metabolic connectivity involving 28 bilateral regions of interest and including regions with altered tracer uptake identified through SPM analysis. As shown in **Figure 7A** and **Table S4,** gain in connections, i.e., present in ALI but not in control animals, was observed in the thalamic connectivity to the amygdala, hippocampus, anterior pretectal nucleus /midbrain, and substantia nigra. Gains in connectivity were also found in the periaqueductal grey/midbrain to substantia nigra, caudate putamen to hippocampus, and cerebellum to amygdala. As shown in **Figure 7B**, these alterations in metabolic connectivity included the thalamus, the hippocampus, and other regions of interest, in which increased [^11^C]PBR28 uptake was identified using SPM analysis at both 24 h and 48 h (**Tables S2 and S3**). Losses in brain metabolic connectivity were also observed in mice with ALI vs controls. These losses included the cerebellar to bed nucleus stria terminalis/extended amygdala, cochlear nuclei/brainstem, and mesencephalic nuclei/midbrain (**Figure 7C**, **Table S5**). Lost connections were also detected from the mesencephalic nuclei/midbrain to caudate putamen, pontine nuclei/pons to cerebellum and hypothalamus, cochlear nuclei/brainstem to bed nucleus stria terminalis/extended amygdala, hippocampus to cerebellum, and substantia nigra/midbrain to cerebellum. As shown in **Figure 7D**, these decreases in brain connectivity in mice with ALI involved regions of interest such as the cerebellum with increased [^11^C]PBR28 uptake predominantly on the left side (hemisphere) **(Tables S2 and S3).** Together, these findings demonstrate that ALI is associated with characteristic alterations in the brain network connectivity that may be driven by increased [^11^C]PBR28 uptake.

## Discussion

Here, using microPET imaging we demonstrate significant brain region-specific alterations indicative of neuroinflammation and altered energy metabolism during ALI in mice.

Brain alterations such as neuroinflammation in ALI/ALF have been attributed to increased levels of ammonia and other neuroactive and neurotoxic substances and pro-inflammatory cytokines impacting brain astrocytes and microglia 3, 14, 15. Circulating IL-6 and other cytokines during peripheral inflammation may enter the brain and alter microglial and astrocyte function, triggering consequent neuroinflammatory responses with significant deleterious impact on the brain 3, 9, 14, 46, 49. There is a link between increased circulating IL-6 levels and the severity of hepatic encephalopathy in patients with ALF 3, 50. Administering a high APAP dose (600 mg/kg) to mice generates sustained hepatocellular damage (studied up to 96h) 33, other features of ALF, and inflammatory alterations 41, which mimic the pathology in patients with severe ALI that progresses into ALF. As in ALF patients with early stages of hepatic encephalopathy 51, a decrease in the cerebral blood flow also is demonstrated in this model as early as 24 h following APAP administration. In addition, circulating IL-6 levels are significantly elevated in these mice and neutralization of IL-6 using an antibody treatment fully restores the cerebral blood flow 41. These observations demonstrate a key mediating role of IL-6 in developing brain alterations in this model of ALI/ALF 41.

Consistent with previous reports 33, 41, we found that administration of APAP (600 mg/kg) resulted in significant hepatocellular damage, elevated ALT and AST, and increased hepatic and serum IL-6 levels at 24 h. In parallel with these peripheral alterations characterizing liver injury and hepatic and systemic inflammation, at 24 h we found increased density of hippocampal microglia, indicative of neuroinflammation. Similar association between peripheral metabolic alterations, increased IL-6 levels and neuroinflammatory alterations was demonstrated at 48 h. Considering previous observations 41 our results suggest that IL-6 may play a role in bridging peripheral inflammation and brain neuroinflammatory changes in mice with APAP induced AL/ALI. However, performing future experiments, including treatments with an IL-6 neutralizing antibody will provide further evidence to establish causality.

Hepatic encephalopathy is a central diagnostic criterion for ALF and one of the principal manifestations of advanced ALI/ALF 13, 52. However, it remains challenging to detect the onset of hepatic encephalopathy clinically. Brain inflammation (neuroinflammation) and altered brain energy metabolism substantially contribute to the development of hepatic encephalopathy in ALF and have an impact on the prognosis and outcome of the disease 1, 3, 14-17. In minimal hepatic encephalopathy, neuroinflammation has been considered a precipitating event that contributes to neurocognitive dysfunction. Our microPET imaging results suggest that important features of hepatic encephalopathy and pathogenesis such as neuroinflammation and alterations in brain energy metabolism, can be detected at relatively early stages (as early as 24 h) of APAP-induced ALI which progresses into ALF.

We used an advantageous conjunction analysis of the microPET imaging 28, which allows for identifying brain regions with overlapping neuroinflammatory (based on [^11^C]PBR28 uptake) and metabolic (based on [^18^F]FDG uptake) alterations in addition to indicating alterations in the brain uptake of each tracer separately. By identifying brain regions where both processes overlap, the conjunction analysis reveals areas of maximal pathological impact that might be prioritized in diagnostic approaches or as targets for therapeutic interventions. The dual-tracer conjunction approach significantly enhances sensitivity, especially when the effects are consistent across individual tracer analyses 53. The value of such integrated, cross-modal analysis is further supported by systematic reviews of concurrent FDG and TSPO PET studies, which highlight its utility in identifying biologically coherent metabolic-inflammatory relationships across conditions 54. Ultimately, this integration not only enhances sensitivity to these coherent biological patterns but also minimizes the risk of tracer-specific false positives or anatomically inconsistent findings that can arise from interpreting each tracer in isolation.

Applying this analysis revealed a significantly increased dual tracer uptake in the thalamus, hippocampus, and habenular nucleus at 24 h and in the thalamus, hypothalamus, and cerebellum at 48 h. These observations were supported by the corresponding individual increases in [^18^F]FDG and [^11^C]PBR28 uptake in these brain areas. In addition, our observations that [^11^C]PBR28 uptake was significantly increased in the hypothalamus, the amygdala, and the left caudate-putamen at 24 h (with no [^18^F]FDG uptake increase) support significant neuroinflammatory responses that are not necessarily associated with increased energy metabolism. One possibility is that changes in microglial/astroglial activation occur early and are then followed by increased metabolism. In addition, the enhanced [^18^F]FDG uptake in the right caudate-putamen at 24 h (with no [^11^C]PBR28 uptake increase) reflects the utility of this tracer in selectively detecting metabolic alterations, which may reflect behavioral or other changes not strictly related to neuroinflammation. Individual analysis also highlighted an increase in either [^18^F]FDG or [^11^C]PBR28 uptake in a different part of the hippocampus and the right caudate-putamen at 48 h, while the conjunction analysis did not show a simultaneous increase in these brain areas.

It should be noted that the observed laterality of tracer uptake may reflect both neurobiological variability following liver injury in animal models or patients and technical variability such as test-retest variation in PET imaging with [^18^F]FDG or [^11^C]PBR28. Differences between conjunction and individual analyses may be expected considering that: 1) the conjunction analysis only captures common brain regions sharing significant changes in uptake for both tracers in individual analysis; and 2) there are large differences in brain-wide signal-to-noise characteristics between PET imaging data for both tracers and each individual tracer. Hence, any region detected in only one tracer or at different locations of the same region would not appear in the conjunction analysis. Moreover, both biological and technical factors, including sample size may lead to lower statistical power in brain mapping analysis with SPM. Because precise alignment of the two datasets requires careful spatial normalization and cross-modal registration to minimize voxel-wise bias, these factors were controlled in our study through standardized preprocessing procedures and quality checks.

The differential impact of ALI on the brain was indicated by the observed decreases in [^18^F]FDG and [^11^C]PBR28 uptake. At 24 h there were only individual tracer uptake decreases affecting different brain areas; [^18^F]FDG uptake was decreased the primary and the supplementary motor cortex, and [^11^C]PBR28 uptake was decreased in the cerebellum. At 48 h we detected overlapping and individual decreases in ^18^F]FDG and [^11^C]PBR28 uptake in the primary somatosensory cortex. These observations are intriguing because alterations in both somatosensory and motor systems have been reported in patients with hepatic encephalopathy 55, which may be linked to the tactile and perceptual discrimination abnormalities observed in these patients 56. In other preclinical studies of APAP intoxication, decreases in locomotor activity have been associated with APAP brain neurotoxicity, possibly affecting areas involved in motor control and regulation 57. Our results support the notion that sensory and motor processing regions of the brain are particularly sensitive to changes induced by APAP-induced ALI. While these observations may be related at least in part to the use of normalized radiotracer uptake in the analysis, further studies would be necessary for adequate neurobiological interpretations.

Until relatively recently, brain [^18^F]FDG uptake, determined using PET, was considered as a proxy of neuronal activity and its changes during pathophysiological states such as neurodegenerative diseases 58. However, microglial activation has also been linked to increased [^18^F]FDG uptake using microPET in mouse models of Alzheimer's disease 59, 60 and experimental neuroinflammatory conditions such as murine amyloidosis 61. A significant correlation between microglial activity and [^18^F]FDG uptake using PET was also observed in patients with neurodegenerative diseases 61. Moreover, the [^18^F]FDG PET signal also reflects astrocyte activity because considerable glucose utilization by astrocytes has also been demonstrated 62. Hence, our results showing region specific alterations in [^18^F]FDG uptake indicative of changes in brain energy metabolism during APAP-induced ALI stem from neuronal, microglial, and astrocytic contributions.

Our results highlight neuroinflammatory and metabolic alterations of APAP induced ALI in main brain regions with regulatory functions that are essential for homeostasis. Notably, the thalamus - affected at both 24 h and 48 h - is a multinuclear structure and a major brain region that receives sensory information. It is reciprocally connected with the cortex, the amygdala, the striatum, and other brain areas and this connectivity is key for mounting a variety of physiological and behavioral responses 63, 64.

We provide further insights into the brain alterations during ALI by leveraging metabolic connectivity analysis of [^18^F]FDG data by adapting and applying a method for metabolic connectivity assessment, previously used in human brain network analysis 40, 65-69. The metabolic connectivity analysis approach we employed has been extensively validated in human neurodegenerative diseases and other neurological disorders. It is based on the principle that correlated metabolic activity between brain regions reflects functional connectivity, which is biologically conserved across mammalian species. Our adaptation to mice involved using a validated Waxholm atlas for anatomical parcellation and bootstrap resampling to ensure statistical robustness of the correlation estimates. We reveal characteristic patterns of gained and lost [^18^F]FDG network connectivity in mice with ALI. For instance, we identify gains in thalamic nuclei metabolic connectivity to multiple regions, including the amygdala and the hippocampus, in which the conjunction and individual SPM analysis also revealed alterations. At this point we can speculate that gains in brain connectivity during ALI could indicate compensatory mechanisms aimed at maintaining brain functionality underlying cognitive, motor, and other important functions. We also demonstrate the dichotomy of brain metabolic connectivity alterations during ALI by observing patterns of significant losses. Importantly, [^11^C]PBR28 increases overlap with [^18^F]FDG in some nodes, defining increased or lost metabolic connectivity during ALI. These observations may reflect the complex interplay between neuroinflammatory and metabolic alterations. Changes in microglia and astrocytes underlying neuroinflammation that can be detected using [^11^C]PBR28 also contribute to the [^18^F]FDG signal. However, neuroinflammation may lead to impaired neuronal function and [^18^F]FDG defined metabolic connectivity. In addition, it is important to note that even increases in [^11^C]PBR28 uptake in nodes (regions of interests) that do not directly overlap with [^18^F]FDG increases may affect metabolic connectivity. While localized, neuroinflammation may affect neuronal function and connectivity associated with other distant nodes, with no changes in the [^11^C]PBR28 uptake.

Future preclinical studies in other conditions primarily driven by peripheral metabolic and inflammatory derangements in which brain dysfunction also is documented will provide insights into whether our findings are specific for ALI/ALF or there are some common elements. For instance, brain neuronal dysfunction and neuropsychiatric manifestations, defined as *sepsis-associated encephalopathy* occur early during sepsis and are associated with increased hospital mortality 8, 70-73. Therefore, applying our approach to characterize “neuroinflammetabolic” signatures and connectivity alterations at early stages of sepsis would be of specific interest. Performing such a study first in preclinical settings is feasible because murine models such as cecal ligation and puncture-induced polymicrobial sepsis, are frequently used to investigate sepsis pathophysiology and its impact on the brain and treatment modalities 74-76.

Performing future clinical studies utilizing this dual tracer PET imaging approach in ALI/ALF and other disorders also is feasible and will be facilitated by the established safety profile of both tracers. [^18^F]FDG is clinically approved and has been used for PET imaging in millions of patients with minimal adverse effects. [^11^C]PBR28 has also been utilized in numerous clinical studies without significant safety concerns. The main challenge in translating this approach is the short half-life of [^11^C], which necessitates rapid production using an on-site cyclotron. There is also the practical consideration of sequential imaging sessions separated by about two hours to allow for [^11^C]PBR28 radioactive decay, which may be more challenging for some severely ill individuals.

## Conclusion

Our results demonstrate, for the first time, brain regional inflammatory and metabolic changes and brain network alterations that can be detected using dual tracer non-invasive microPET imaging and advanced analysis in mice at relatively early stages of high dose APAP induced ALI/ALF. These findings provide a preclinical platform for non-invasively diagnosing key signs of hepatic encephalopathy. This is important because targeted therapies can be applied in a timely manner and further PET evaluation can be utilized to assess treatment efficacy. Our approach also is applicable to brain assessments in the context of other disorders “classically” characterized by peripheral immune and metabolic dysregulation, with the goal of applying timely therapeutic interventions.

## Supplementary Material

Supplementary figures and tables.

## Abbreviations

ALI: acute liver injury
ALF: acute liver failure
APAP: acetaminophen (N-acetylp-aminophenol
PET: positron emission tomography
ALT: alanine aminotransferase
AST: aspartate aminotransferase
[^11^C]PBR28: [^11^C]-peripheral benzodiazepine receptor
[^18^F]FDG: [^18^F]-fluoro-2-deoxy-2-D-glucose
IAW: Inveon Acquisition workflow
PMOD: Pixel-Wise Modeling
SPM: statistical parametric mapping
VOI: volume-of-interest
