# Supplementary material for: Non-invasive identification of brain signatures of acute liver injury
Source: Theranostics. 2026 Jan 21;16(8):4058–75. doi: 10.7150/thno.127621 (PMC12905780; doi:10.7150/thno.127621)
Supplement: Supplementary file 1 — Supplementary figures and tables. [file thnov16p4058s1.pdf]

Supplementary figures and tables

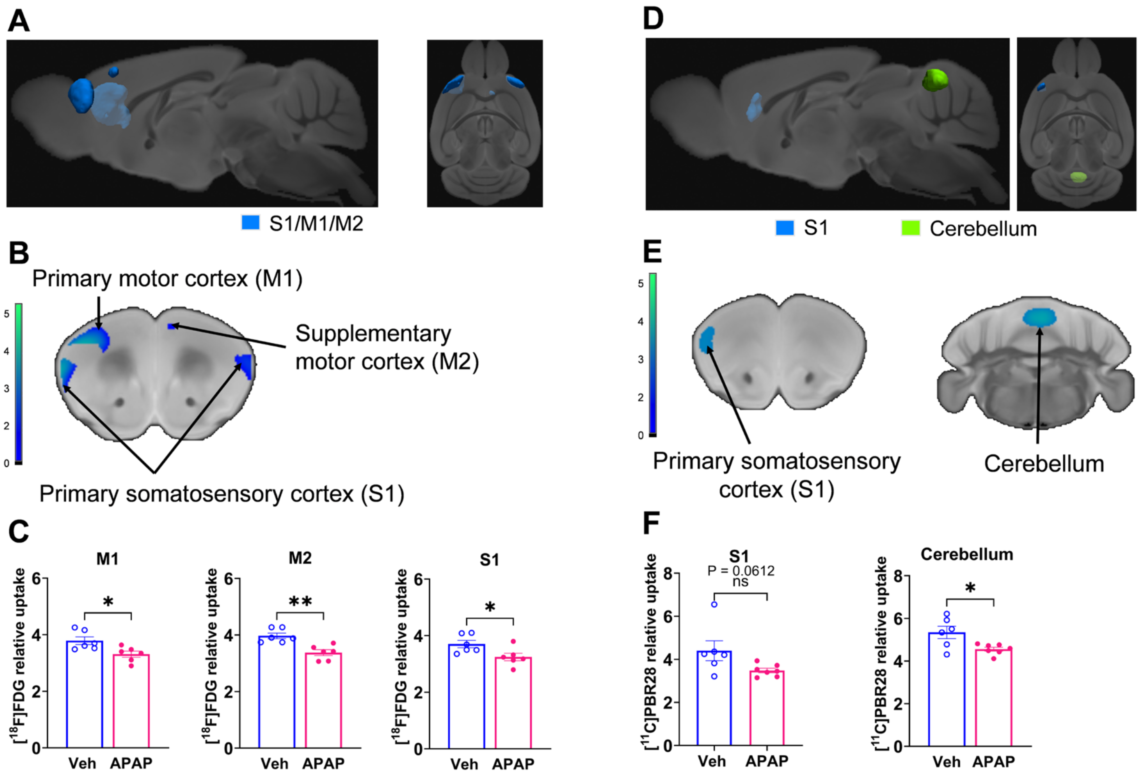

Figure S1. Brain regions with decreased individual [^{11}C]PBR28 and [^{18}F]FDG uptake at 24 h during ALI. (**A**) Cumulative [^{18}F]FDG uptake decreases (statistically significant clusters at P < 0.01) in the primary somatosensory cortex and the primary and supplementary motor cortices in APAP administered vs control mice as 3D clusters overlaid on brain sagittal and axial MRI templates; and (**B)** overlaid on brain coronal MRI templates (color bar represents t-value height, cutoff threshold T = 2.4). (**C)** Post-hoc analysis of [^{18}F]FDG tracer uptake in the primary motor cortex (M1) (**P* = 0.021; Student's t test), in the secondary motor cortex (M2) (***P* = 0.002; Student's t test), and in the primary somatosensory cortex (S1) (**P* = 0.034; Student's t test) (n = 6). (**D**) 3D clusters of cumulative decreases in [^{11}C]PBR28 uptake (*P* < 0.01) in the primary somatosensory cortex and the cerebellum in APAP administered vs control mice. (**E**) Cumulative decreases in [^{11}C]PBR28 uptake (*P* < 0.01) on brain coronal MRI brain templates (color bar represents t-value height, cutoff threshold T = 2.4). (**F)** Post-hoc analysis of [^{11}C]PBR28 uptake in the primary somatosensory cortex (S1) (*P* = 0.061; Student's t test) and the cerebellum (*P* = 0.018; Student's t test) (n = 6,7). Data are presented as individual mouse data points with mean ± SEM.

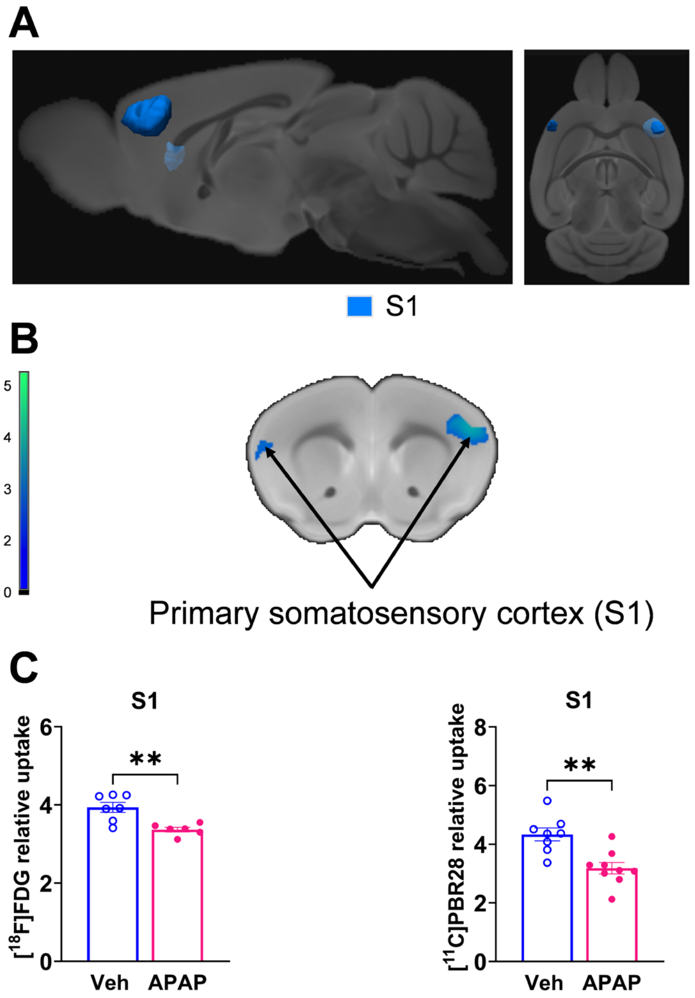

Figure S2. Brain regions with decreased overlapping [^{11}C]PBR28 and [^{18}F]FDG uptake at 48 h during ALI. (**A**) Cumulative dual tracer uptake decreases (statistically significant clusters at P < 0.01) in the primary somatosensory cortex in APAP administered vs control mice as 3D clusters overlaid on sagittal and axial MRI templates; and (**B)** overlaid on coronal MRI templates (color bar represents t-value height, cutoff threshold T = 2.4) (**C**) Post-hoc analysis of [^{18}F]FDG (***P* = 0.003; Student's *t*-test) and [^{11}C]PBR28 (***P* = 0.001; Students *t*-test) decreases in the primary somatosensory cortex of the same groups of mice. (Controls, n = 7,8; APAP treated n = 6,9) Data are presented as individual mouse data points with mean ± SEM.

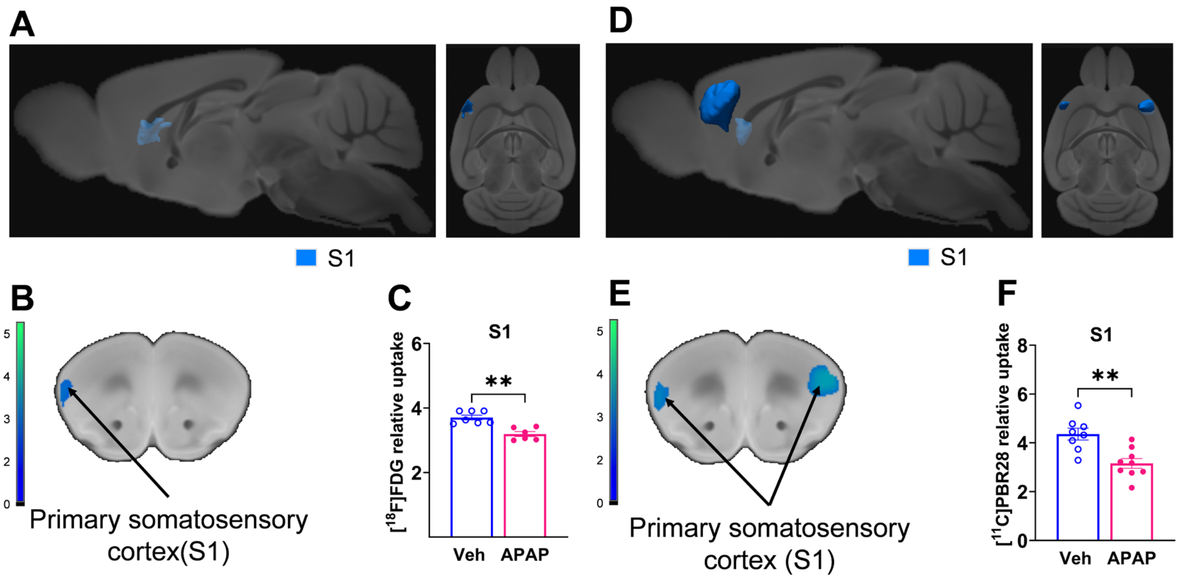

Figure S3. Brain regions with decreased individual [^{11}C]PBR28 and [^{18}F]FDG uptake at 48 h during ALI. (**A**) Cumulative [^{18}F]FDG uptake decreases (statistically significant clusters at $P < 0.01$) in the primary somatosensory cortex in APAP administered vs control mice as 3D clusters overlaid on brain sagittal and axial MRI templates; and (**B**) affected areas overlaid on brain coronal MRI templates (color bar represents t-value height, cutoff threshold T = 2.4). (**C**) Post-hoc analysis of [^{18}F]FDG tracer uptake in the primary somatosensory cortex (S1) (***P* = 0.001; Mann-Whitney test). (Controls, n = 7; APAP treated n = 6) (**D**) 3D clusters of cumulative decreases in [^{11}C]PBR28 uptake ($P < 0.01$) in the primary somatosensory cortex in APAP administered vs control mice. (**E**) Cumulative decreases in [^{11}C]PBR28 uptake ($P < 0.01$) on brain coronal MRI brain templates (color bar represents t-value height, cutoff threshold T = 2.4). (**F**) Post-hoc analysis of [^{11}C]PBR28 uptake in the primary somatosensory cortex (S1) (***P* = 0.002; Student's t test). (Controls, n = 8; APAP treated n = 9). Data are presented as individual mouse data points with mean ± SEM.

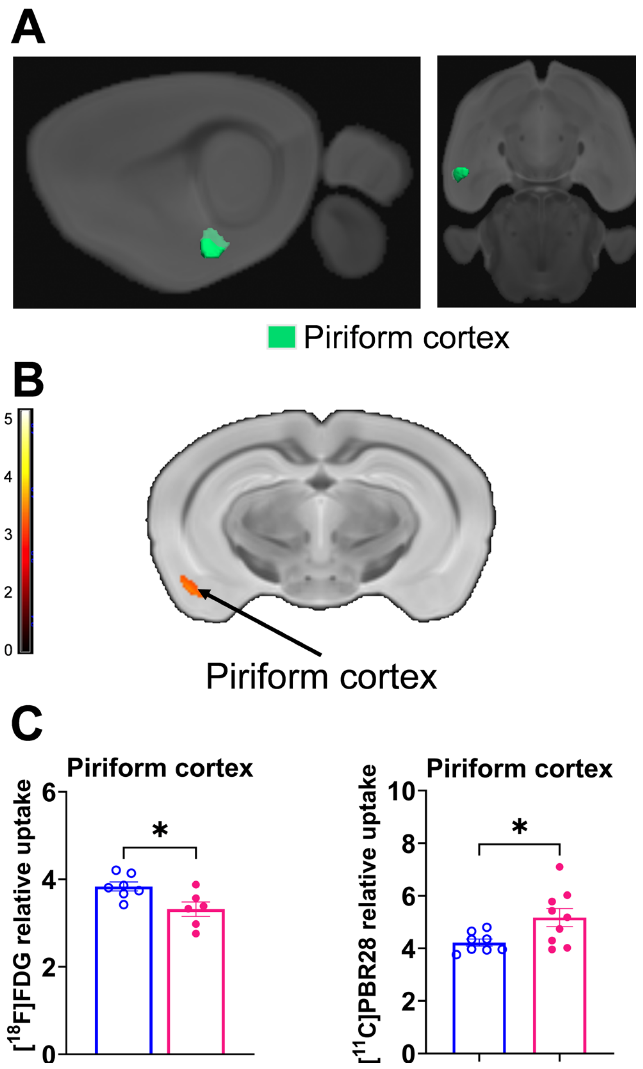

Figure S4. Brain regions with an individual [^{18}F]FDG uptake decrease and [^{11}C]PBR28 increase at 48 h during ALI. (**A**) Cumulative [^{18}F]FDG uptake decrease and [^{11}C]PBR28 uptake increase in the piriform cortex in APAP administered vs control mice as 3D clusters overlaid on brain sagittal and axial MRI templates; and (**B**) overlaid on brain coronal MRI templates (color bar represents t-value height, cutoff threshold T = 2.4). (**C**) Decreased [^{18}F]FDG uptake in the piriform cortex (post-hoc analysis; **P* = 0.018; Student's t test) and increased [^{11}C]PBR28 uptake in the piriform cortex (post-hoc analysis; **P* = 0.028; Student's t test). (Controls, n = 7,8; APAP treated n = 6,9) Data are presented as individual mouse data points with mean ± SEM.

Table S1. Regions of interest (ROI) utilized in network analysis using the Waxholm (WHS) atlas [1].

ROI #	Brain regions	Abbreviation (WHS2012)
1	Sensorimotor cortex*/Neocortex	S1/M1, M2
2	Brainstem	SpC
3	Thalamus	Th
4	Superior colliculus (Midbrain)	SC
5	Inferior colliculus	IC
6	Periaqueductal gray/Midbrain	PAG
7	Septal nuclei/Limbic-frontal	Sept
8	Ventral thalamic nuclei/Thalamus	Th
9	Pontine nuclei/Pons	Pons
10	Substantia nigra/Midbrain	SN
11	Interpeduncular nucleus/Midbrain	MBr-u (unsegmented)
12	Globus pallidus	GP
13	Mesencephalic nuclei/Midbrain	MBr-u (unsegmented)
14	Laterodorsal nucleus/Thalamus	Th
15	Medial geniculate nucleus/Thalamus	Th
16	Anterior pretectal nucleus/Midbrain	MBr-u (unsegmented)
17	Caudate/Putamen	Str
18	Hippocampus	**HiP
19	Lateral geniculate nucleus/Thalamus	Th
20	Amygdala	Amg

21	Hypothalamus	Hy
22	Nucleus accumbens/Striatum	Str
23	Cochlear nuclei/Brainstem	SpC
24	Cerebellum	Cb
25	Piriform cortex	**PIR
26	Preoptic area/Anterior hypothalamus	Hy
27	Bed nucleus stria terminalis /extended amygdala	**BST
28	Ventral pallidum	**PALv

*The sensorimotor cortex is defined using the neocortex atlas [2]

**Interactive Allen Mouse Brain Atlas abbreviation. https://atlas.brain-map.org/ (2011).

Table S2. Specific brain regions with significant [^{18}F]FDG and [^{11}C]PBR28 uptake increases* at 24 h

Radiotracer<br>Brain region	Brain coordinates**				
	Mediolateral (mm)	Anteroposterior (mm)	Dorsoventral (mm)	Relative uptake values (Vehicle)	Relative uptake values (APAP)
**Dual [^{18}F]FDG and [^{11}C]PBR28 uptake**					
Anterior nuclei of thalamus	1.3	-2.9	-2.9	[^{18}F]FDG = 4.43 ± 0.11<br>[^{11}C]PBR28 = 3.01 ± 0.26	[^{18}F]FDG = 4.91 ± 0.16<br>[^{11}C]PBR28 = 3.80 ± 0.04
Hippocampus (CA3)	3.1	-2.8	-3.6	[^{18}F]FDG = 4.05 ± 0.05<br>[^{11}C]PBR28 = 3.61 ± 0.07	[^{18}F]FDG = 4.37 ± 0.12<br>[^{11}C]PBR28 = 4.22 ± 0.02

Medial habenular nucleus	0.5	-1.0	-2.7	[^{18}F]FDG = 4.26 ± 0.05 [^{11}C]PBR28 = 3.82 ± 0.12	[^{18}F]FDG = 4.58 ± 0.12 [^{11}C]PBR28 = 4.33 ± 0.08
**[^{18}F]FDG uptake**					
Posterior thalamic nuclear group	-0.5	-2.6	-3.8	4.39 ± 0.09	4.83 ± 0.13
Lateral habenular nucleus	-0.5	-1.1	-2.8	4.32 ± 0.07	4.78 ± 0.13
Hippocampus (CA3)	3.1	-2.8	-3.6	4.06 ± 0.06	4.38 ± 0.12
Caudate - Putamen	2.2	0.7	-3.8	4.04 ± 0.06	4.36 ± 0.11
**[^{11}C]PBR28 uptake**					
Post-thalamic nuclear group	1.5	-2.8	-3.4	2.95 ± 0.27	3.76 ± 0.03
Hypothalamic supramammillary area	0.4	-2.8	-4.7	3.54 ± 0.27	4.28 ± 0.11

Hippocampus (CA1/CA2)	2.9	-2.8	-3.0	3.39 ± 0.07	4.01 ± 0.03
Central amygdaloid nucleus	2.3	-1.1	-5	3.48 ± 0.14	4.10 ± 0.10
Caudate – Putamen (near external capsule)	-2.6	-0.5	-2.4	3.13 ± 0.12	3.62 ± 0.07
Lateral habenular nucleus	0.5	-1.1	-2.7	3.23 ± 0.23	3.98 ± 0.03

*Increased tracer uptake was identified using statistical parametric mapping (SPM) with $P < 0.01$ in the conjunction analysis and in the individual tracer uptake analysis (with an extent threshold of t = 200 voxels), comparing vehicle administered mice vs APAP administered mice.

**According to [3]

Table S3. Specific brain regions with significant [^{18}F]FDG and [^{11}C]PBR28 uptake increases* at 48 h

Radiotracer<br>Brain region	Brain coordinates**			Relative uptake values (Saline)	Relative uptake values (APAP)
	Mediolateral (mm)	Anteroposterior (mm)	Dorsoventral (mm)		
**Dual [^{18}F]FDG and [^{11}C]PBR28 uptake**					
Parafascicular thalamic nucleus	-0.5	-2.6	-3.4	[^{18}F]FDG = 4.46 ± 0.08<br>[^{11}C]PBR28 = 3.01 ± 0.09	[^{18}F]FDG = 5.11 ± 0.11<br>[^{11}C]PBR28 = 4.04 ±0.4
Dorsomedial hypothalamic nucleus	-0.2	-1.7	-5.0	[^{18}F]FDG = 3.84 ± 0.10<br>[^{11}C]PBR28 = 3.00 ± 0.18	[^{18}F]FDG = 4.52 ± 0.18<br>[^{11}C]PBR28 = 4.17 ± 0.26

8th cerebellar lobule	0.2	-7.0	-2.9	[18F]FDG = 4.78 ± 0.19 [11C]PBR28 = 4.03 ± 0.19	[18F]FDG = 5.69 ± 0.26 [11C]PBR28 = 5.43 ± 0.17
**[18F]FDG uptake**					
Anterior nuclei of thalamus	0.8	-2.7	-2.4	4.37 ± 0.08	4.84 ± 0.12
Lateral hypothalamic area	-0.7	-2.7	-4.6	3.86 ± 0.09	4.53 ± 0.15
Hippocampus (Radiatum layer)	-0.5	-1.1	-2	4.16 ± 0.04	4.61 ± 0.10
1º fissure Cerebellum	-1.9	-5.4	-2.4	4.56 ± 0.10	5.38 ± 0.28
Caudate - Putamen	2.0	-0.2	-3.0	3.89 ± 0.07	4.30 ± 0.10
**[11C]PBR28 uptake**					
Superior thalamic radiation	1.9	-1.9	-2.6	2.92 ± 0.15	3.51 ± 0.18

Lateral hypothalamic area	0.7	-1.6	-5.2	3.05 ± 0.19	4.26 ± 0.26
Hippocampal - External capsule (entorhinal)	-3.7	-3.6	-4.1	3.97 ± 0.22	4.99 ± 0.39
Caudate - Putamen	-2.7	-0.8	-2.7	3.06 ± 0.10	3.98 ± 0.31
8th cerebellar lobule	-0.2	-7.0	-2.9	4.01 ± 0.19	5.49 ± 0.17

*Increased tracer uptake was identified using statistical parametric mapping (SPM) with $P < 0.01$ in the conjunction analysis and in the individual tracer uptake analysis (with an extent threshold of t = 200 voxels), comparing vehicle administered mice vs APAP administered mice.

**According to [3]

Table S4. Gained connections in the metabolic network in mice with acute liver injury relative to control mice.

ROI 1*	ROI 2*	R (Vehicle)**	R (APAP)**	*P*-value
Laterodorsal nucleus/Thalamus (L)	Amygdala (L)	0.08	-0.76	1.7E-71
Laterodorsal nucleus/Thalamus (R)	Anterior pretectal nucleus/Midbrain (R)	0.39	0.90	1.5E-70
Laterodorsal nucleus/Thalamus (R)	Lateral geniculate nucleus/Thalamus (R)	-0.03	0.86	2.8E-51
Laterodorsal nucleus/Thalamus (R)	Medial geniculate nucleus/Thalamus (R)	-0.12	0.88	1.8E-103
Ventral thalamic nuclei/Thalamus (R)	Hippocampus (R)	0.04	0.74	9.9E-41
Ventral thalamic nuclei/Thalamus (R)	Laterodorsal nucleus/Thalamus (R)	-0.17	0.89	7.8E-99
Ventral thalamic nuclei/Thalamus (L)	Substantia nigra/ Midbrain (L)	0.00	0.78	2.5E-35
Mesencephalic nuclei/Midbrain (L)	Laterodorsal nucleus/Thalamus (L)	-0.01	0.71	1.2E-42
Mesencephalic nuclei/Midbrain (R)	Laterodorsal nucleus/Thalamus (R)	0.01	0.75	7.8E-43
Periaqueductal gray/ Midbrain (R)	Mesencephalic nuclei/ Midbrain (R)	0.12	0.72	3.5E-39
Periaqueductal gray/Midbrain (R)	Substantia nigra/Midbrain (R)	0.13	0.73	2.1E-26

Thalamus (R)	Hippocampus (R)	0.27	0.77	4.1E-28
Thalamus (R)	Laterodorsal nucleus/Thalamus (R)	0.23	0.84	1.6E-51
Brainstem (L)	Cochlear nuclei (L)	0.15	0.74	5.7E-51
Caudate/Putamen (R)	Hippocampus (R)	0.25	0.83	2.6E-47
Cerebellum (L)	Amygdala (R)	0.37	-0.85	9.2E-103
Medial geniculate nucleus/Thalamus (R)	Hippocampus (R)	0.00	0.79	1.7E-53
Substantia nigra/ Midbrain (L)	Medial geniculate nucleus/Thalamus (L)	0.30	0.81	5.0E-37

* Changes in connectivity between nodes designated as ROI 1 (region of interest 1) and ROI 2 (region of interest 2).

** Pearson correlation coefficient (R) defines the strength (connectivity value) of the edge linking ROI 1 and ROI 2 for each group (APAP or vehicle) reported. 100 R values (bootstrap iterations) were compared between the two groups, producing a *P*-value (Student's t-test) for each connection. The median R value of 100 bootstrap iterations was shown for each connection.

Table S5. Lost connections in the metabolic network in mice with acute liver injury relative to control mice.

ROI 1*	ROI 2*	R (Vehicle)**	R (APAP)**	*P*-value
Cerebellum (L)	Bed nucleus stria terminalis /extended amygdala-(R)	-0.75	-0.21	7.1E-33
Cerebellum (L)	Cochlear nuclei/Brainstem (R)	-0.78	-0.23	3.1E-26
Cerebellum (L)	Mesencephalic nuclei/Midbrain (R)	-0.73	-0.18	3.2E-36
Mesencephalic nuclei/ Midbrain (L)	Caudate/Putamen (L)	-0.81	-0.22	5.7E-49
Mesencephalic nuclei/ Midbrain (L)	Cerebellum (L)	-0.75	-0.16	6.7E-43
Pontine nuclei/Pons (L)	Cerebellum (L)	-0.80	-0.10	4.9E-47
Pontine nuclei/Pons (L)	Hypothalamus (L)	0.71	0.20	6.5E-33
Cochlear nuclei/ Brainstem (L)	Bed nucleus stria terminalis /extended amygdala (L)	0.80	-0.13	6.6E-75
Hippocampus (L)	Cerebellum (L)	0.74	-0.12	2.5E-70
Substantia nigra/ Midbrain (L)	Cerebellum (L)	-0.78	-0.02	1.2E-34

* Changes in connectivity between nodes designated as ROI 1 (region of interest 1) and ROI 2 (region of interest 2).

** Pearson correlation coefficient (R) defines the strength (connectivity value) of the edge linking ROI 1 and ROI 2 for each group (APAP or vehicle) reported. 100 R values (bootstrap iterations)

were compared between the two groups, producing a *P*-value (Student's t-test) for each connection. The median R value of 100 bootstrap iterations was shown for each connection.

References

1. Johnson GA, Badea A, Brandenburg J, Cofer G, Fubara B, Liu S, et al. Waxholm space: an image-based reference for coordinating mouse brain research. NeuroImage. 2010; 53: 365-72.
2. Ullmann JF, Watson C, Janke AL, Kurniawan ND, Reutens DC. A segmentation protocol and MRI atlas of the C57BL/6J mouse neocortex. NeuroImage. 2013; 78: 196-203.
3. Paxinos G, Franklin KB. Paxinos and Franklin's the mouse brain in stereotaxic coordinates: Academic Press; 2019.
